# Streptonigrin at low concentration promotes heterochromatin formation

**DOI:** 10.1038/s41598-020-60469-6

**Published:** 2020-02-26

**Authors:** Andre C. Loyola, Kevin Dao, Robin Shang, Lin Zhang, Pranabananda Dutta, Cody Fowler, Jinghong Li, Willis X. Li

**Affiliations:** Department of Medicine, University of California San Diego, La Jolla, CA 92093 USA

**Keywords:** Phenotypic screening, Drug development

## Abstract

Heterochromatin is essential for regulating global gene transcription and protecting genome stability, and may play a role in tumor suppression. Drugs promoting heterochromatin are potential cancer therapeutics but very few are known. In order to identify drugs that can promote heterochromatin, we used a cell-based method and screened NCI drug libraries consisting of oncology drugs and natural compounds. Since heterochromatin is originally defined as intensely stained chromatin in the nucleus, we estimated heterochromatin contents of cells treated with different drugs by quantifying the fluorescence intensity of nuclei stained with Hoechst DNA dye. We used HeLa cells and screened 231 FDA-approved oncology and natural substance drugs included in two NCI drug libraries representing a variety of chemical structures. Among these drugs, streptonigrin most prominently caused an increase in Hoechst-stained nuclear fluorescence intensity. We further show that streptonigrin treated cells exhibit compacted DNA foci in the nucleus that co-localize with Heterochromatin Protein 1 alpha (HP1α), and exhibit an increase in total levels of the heterochromatin mark, H3K9me3. Interestingly, we found that streptonigrin promotes heterochromatin at a concentration as low as one nanomolar, and at this concentration there were no detectable effects on cell proliferation or viability. Finally, in line with a previous report, we found that streptonigrin inhibits STAT3 phosphorylation, raising the possibility that non-canonical STAT function may contribute to the effects of streptonigrin on heterochromatin. These results suggest that, at low concentrations, streptonigrin may primarily enhance heterochromatin formation with little toxic effects on cells, and therefore might be a good candidate for epigenetic cancer therapy.

## Introduction

Heterochromatin is essential for chromosomal compaction and transcriptional silencing, and more recently it has been shown to play a role in animal longevity and tumor suppression^1–4^. Indeed, cellular differentiation is associated with increases in heterochromatin levels^5–8^, and a hallmark of cancer development is dedifferentiation, which is accompanied by loss of heterochromatin^9^. Many cancers are associated with heterochromatin loss and derepression of satellite repeats^10–12^, and cancer development occurs in cells that have decreased levels of heterochromatin or are unable to form new heterochromatin^13,14^. Consistent with these findings, we have shown that heterochromatin is essential for maintaining genome stability^15^, and suppresses tumor growth^16^. Thus promoting heterochromatin formation may represent an epigenetic cancer therapy.

We have previously developed a screening method for identifying compounds that promote heterochromatin formation using *Drosophila*^17^. While our assay using *Drosophila* offers a sensitive way to screen for drugs *in vivo*, it is relatively tedious and subjective, requiring the observer to assign an eye color score to each fly under the microscope. With an interest in developing a high throughput method for a streamlined drug screening process, we designed a simple screen using HeLa cells based on fluorescence intensity of the DNA stain Hoechst.

Hoechst stains are blue fluorescent DNA dyes soluble in water and in organic solvents such as dimethyl sulfoxide (DMSO)^18^. Hoechst 33258 and Hoechst 33342 are most commonly used DNA dyes with similar excitation-emission spectra. Both dyes are excited at around 350 nm wave length and emit blue-cyan light that peaks around 461 nm^19^. They bind to double-stranded DNA at minor groove with enhanced fluorescence^20^. DNA compaction, as occurs in heterochromatin formation, will result in an increase in concentration of DNA-bound Hoechst dye and thereby an increase in fluorescence. Indeed, heterochromatin is originally defined as nuclear regions with intensely stained by DNA dye^1–3^. Another commonly used fluorescent DNA stain is 4′,6-diamidino-2-phenylindole (DAPI), which shares similar properties with Hoechst but is less membrane permeant and therefore it is not usually used for live cell staining^21^.

Here, we describe the use mammalian cells with Hoechst stain to screen for heterochromatin-promoting compounds. Since Hoechst stain can be used in live cells and its fluorescence can be imaged on a compound microscope, our screen should be applicable for large scale high throughput screening for heterochromatin promoting drugs toward developing a new category of epigenetic cancer therapeutics.

Our screen identified streptonigrin (SN, CAS no. 3930-19-6), among the 231 oncology and natural substance drugs screened, as the most potent drug in promoting heterochromatin formation. Streptonigrin is an aminoquinone antitumor antibiotic isolated from *Streptomyces flocculus* and has been reported to induce DNA breaks, and it has been previously used as a cancer chemotherapy drug but has been mostly discontinued due to its strong cytotoxic effects^22^. However, these trials were done at micromolar to millimolar concentrations. We have tested the effects of low concentrations streptonigrin treatment in this study and found that at these concentrations (nanomolar level), streptonigrin can enhance heterochromatin formation with little toxic effects on cells. Our results suggest that low concentration streptonigrin might be useful for epigenetic cancer therapy by increasing heterochromatin formation.

## Results

### A cell-based screen to identify heterochromatin-promoting drugs

In order to develop a method appropriate for high throughput screening for compounds that promote heterochromatin formation, we sought to use cell-based imaging, in which the fluorescent intensities of cells in multi-well plates treated with different molecules can be simultaneously recorded using a fluorescent microscope.

We decided to use Hoechst 33342, a membrane-permeable fluorescent DNA dye, to estimate the levels of heterochromatin, which was originally defined as nuclear materials intensely stained by DNA dyes due to its tight packing^1–4^. We chose Hoechst over DAPI because Hoechst is membrane permeable and can be used to stain live cells whereas DAPI can only stain fixed cells. Among Hoechst stains, Hoechst 33342 is more membrane permeant and is better suited than Hoechst 33258 for live staining^23^. The rationale of the screening method is, if a particular compound promotes heterochromatin formation, treating cells with this compound will result in higher fluorescence in cells stained with Hoechst.

For small-molecule compounds, we obtained two drug libraries from the National Cancer Institute (NCI) Developmental Therapeutics Program (DTP) – the Oncology Set IV library, containing 114 FDA approved oncology drugs, and the Natural Products Set III library, consisting of 117 natural compounds that were chosen from the DTP’s repository of 140,000 compounds based on their origin as a natural product, purity, structural diversity, and availability of the compound (Table S1).

To screen for heterochromatin promoting compounds, we seeded HeLa cells at a density of 7,000 cells/well in 96 well plates. After 24 hours of growth, cells in each well were treated with 10 μM concentration of a compound for 4 hours and were then fixed and stained with Hoechst 33342 to reveal the nuclei and chromatin conformation and photographed on a fluorescence microscope. We chose to fix cells in the initial screening in order to allow sufficient time for taking high-resolution images of treated cells for morphological studies, which cannot be done synchronously for 96 wells on a compound microscope without automation.

**Figure 1:**
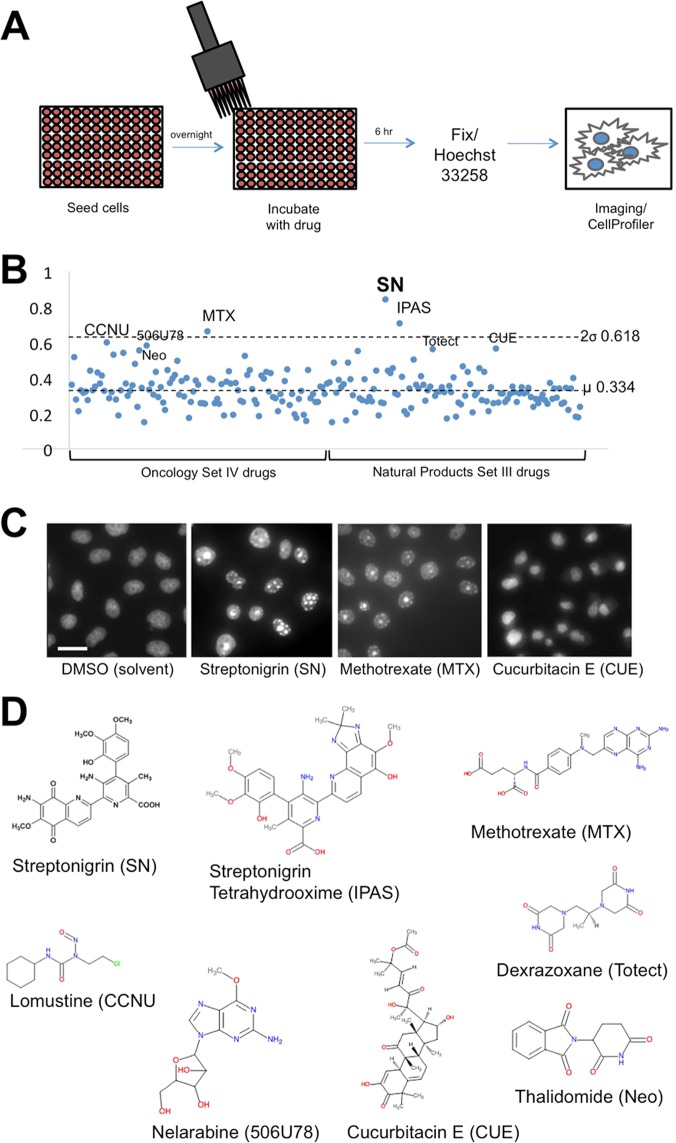
Identification of streptonigrin as promoting heterochromatin formation. (**A**) Schematic illustration of the drug screening method. HeLa cells were seeded into 96 well plates at a density of 7,000 cells/well and left to adhere overnight. Cells in each well were then treated with 10 μM of a compound for 4 hours, and were then fixed with 3.7% formaldehyde and stained with Hoechst 33342. Cells were imaged and analyzed with CellProfiler to obtain fluorescence intensities and to observe the nuclear morphology. (**B**) Scatter plot of mean fluorescence intensities of HeLa cells treated with drugs in the oncology and natural product drug libraries. (**C**) Fluorescence microscopy images of HeLa cells treated 10 μM of the indicated drugs or solvent (DMSO) for 4 hours. Scale bar = 5 μm. (**D**) Chemical structures of the hit drugs.

We analyzed the images of the cells in each well with the open-source software CellProfiler, which is capable of identifying and quantifying biological features of cells in images^24^. We used a built-in intensity measurement module in CellProfiler and obtained the mean fluorescence intensity for each drug (Fig. 1A; Fig. S1).

We screened a total number of 231 compounds included in the two drug libraries as described above. The mean (μ) nuclear fluorescence intensity for all the drugs is 0.334 (normalized arbitrary units) with a standard deviation (σ) of 0.142 (Fig. 1B). For the ones with significantly higher nuclear fluorescence intensities, defined as greater than 2 σ (or >0.618), we further observed the nuclear morphology of the cells through fluorescence microscopy to confirm that these compounds indeed induced more heterochromatin formation rather than cell death, which also results in brighter fluorescence^24^. An increase in heterochromatin is seen as the appearance of multiple foci enriched in Hoechst-stained nuclei (Fig. 1C). On the other hand, dying cells show chromatin margination, nuclear fragmentation and/or condensation^25^ (Fig. S2), and these cells eventually die and detach from the culture plates. These analyses allowed for identification of compounds that may promote DNA compaction or heterochromatin formation in cultures cells.

### Streptonigrin promotes heterochromatin formation in cultured cells

Out of the 231 compounds we screened, one drug, streptonigrin (SN, CAS no. 3930-19-6), was identified as inducing the most dramatic increase in fluorescent intensity of Hoechst-stained cells accompanied by a change to a multi-foci nuclear morphology. The mean fluorescence intensity of HeLa cells treated with streptonigrin is 0.840, which is greater than 3 σ from the mean (Fig. 1B; Table 1; Table S1). Consistent with this interpretation, methotrexate which we have previously identified as a heterochromatin-promoting drug^17^, also caused an increase in fluorescence of treated cells (Fig. 1B; Table 1). HeLa cells treated with these drugs indeed exhibit a general increase in brightness of Hoechst stain and formation of multiple nuclear foci compared to DMSO-only treated controls, indicative of an increase in heterochromatin in these cells, as shown representatively for streptonigrin, methotrexate, and cucurbitacin E (Fig. 1C).

**Table 1:** List of top drug hits with high fluorescence intensity.

Name	Short Name	NSC No.	Intensity
Streptonigrin	SN	45383	0.8401
Streptonigrin tetrahydrooxime	IPAS	62709	0.7085
Methotrexate	MTX	740	0.6636
Lomustine	CCNU	79037	0.5983
Nelarabine	506U78	686673	0.5820
Cucurbitacin E	CUE	106399	0.5603
Dexrazoxane	Totect	169780	0.5532
Thalidomide	Neo	66847	0.5425
Carmustine	BCNU	409962	0.5311
Tamoxifen citrate	TMX	180973	0.5236
Allopurinol	HPP	1390	0.5171
Daunorubicin	DNRN	82151	0.5037
Dacarbazine	DTIC	45388	0.4420

Other compounds that caused an increase in fluorescent intensity of treated cells include Streptonigrin tetrahydrooxime, Lomustine, Nelarabine, Cucurbitacin E, Dexrazoxane, Thalidomide, Carmustine, Tamoxifen citrate, Allopurinol, Daunorubicin, and Dacarbazine (Fig. 1B; Table 1). Most of these drugs have one or more aromatic rings in their chemical structures (Fig. 1D), which may contribute to their ability to cause an increase in heterochromatin levels.

**Figure 2:**
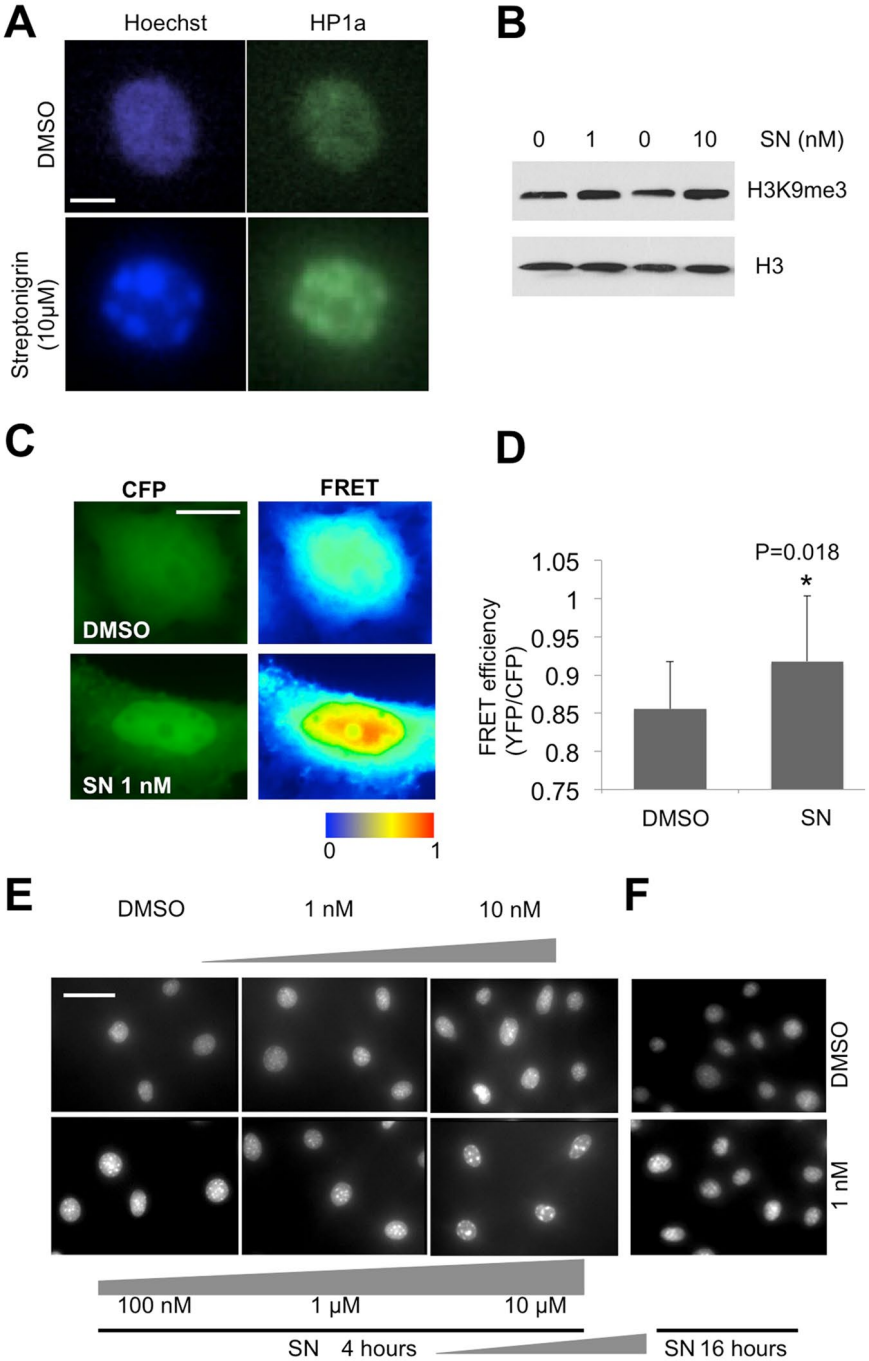
Streptonigrin promotes heterochromatin dependent on time and concentration. (**A**) HeLa cells were treated with solvent only (DMSO) or 10 μM streptonigrin for 4 hours and then were fixed and stained with Hoechst (blue) and primary antibodies against HP1α (green). (**B**) HeLa cells treated the indicated concentrations of streptonigrin or solvent (DMSO) only for 4 hours were homogenized and the total protein was subjected to SDS-PAGE and blotted with antibodies specific for H3K9me3 or H3. Note the increased levels of H3K9me3 after cells were treated with 1 or 10 nM of streptonigrin. Scale bar = 2 μm. (**C**,**D**) HeLa cells were transfected with a heterochromatin FRET sensor, consisting of an HP1-H3 peptide with CFP and YFP. (**C**) Representative FRET images of cells treated for 4 h with or without 1 nM streptonigrin (SN). (**D**) Mean FRET efficiency (YFP/CFP fluorescence ratio) with standard deviations was calculated for treated or untreated cells. * indicates p < 0.05 in Student’s t-test. Scale bar = 2 μm. (**E**) 3T3 cells live stained with DNA stain Hoechst 33342. Cells were treated with log concentrations of streptonigrin or DMSO for 4 hours and visualized with a fluorescence microscope. Scale bar = 10 μm. (**F**) Fluorescence microscopy of 3T3 cells live stained with DNA stain Hoechst 33342 after the cells were treated with 1 nM of streptonigrin or solvent (DMSO) for 16 hours.

To further verify that the increase in fluorescence intensity induced by streptonigrin correlates with an increase in heterochromatin levels, we examined other markers of heterochromatin. We first performed immunostaining with antibodies specific for Heterochromatin Protein 1alpha (HP1α), a central component of heterochromatin^1–4^. In streptonigrin-treated HeLa cells, we observed the co-localization of HP1α and DNA foci enriched with Hoechst (Fig. 2A), confirming that these foci correspond to heterochromatin.

Secondly, we examined histone H3 in total protein extracts of HeLa cells treated with streptonigrin or solvent control, and found that streptonigrin treatment indeed resulted in an increase in the levels of heterochromatin mark, H3K9me3, as revealed by Western blotting with anti-H3K9me3 antibodies (Fig. 2B). Third, we used a fluorescence resonance energy transfer (FRET)-based heterochromatin sensor^26–28^ to assess changes in heterochromatin levels in cells treated with streptonigrin. The FRET heterochromatin sensor consists of fusion peptides of H3-YFP and HP1-CFP, which has been previously used as a robust readout of H3K9me3 levels in cells expressing the fusion peptides^26–28^. Methylation of H3 to form H3K9me3 induces HP1 binding, resulting in FRET between YFP and CFP. Indeed, we found that treating HeLa cells with streptonigrin as low as 1 nM for 4 hours caused a dramatic increase in global heterochromatin levels, as indicated by the FRET sensor (Fig. 2C,D). These results support the notion that streptonigrin promotes heterochromatin formation in cells.

### Streptonigrin induces heterochromatin formation in a concentration and time-dependent manner

Next, we wanted to investigate the concentration and time-dependent effects of streptonigrin on heterochromatin organization. For this purpose and to test a different cell type, we decided to use NIH 3T3 cells, an immortalized mouse fibroblast cell line, which exhibits easily discernible pericentric heterochromatin foci and is more sensitive for observing changes in nuclear morphology^29^. The ability to observe live cells without fixation shortens experimental time and procedure and is advantageous for high throughput screening.

To observe the drug effect on live cells, we seeded NIH 3T3 cells into 24 well plates at a density of 50,000 cells/well, treated with different concentrations of streptonigrin and live stained with DNA stain Hoechst 33342 in order to observe nuclear morphology. 3T3 cells were treated with 1 nM, 10 nM, 100 nM, 1 uM, 10 uM concentrations of streptonigrin or DMSO for 4 hours. We live stained the cells and observed a concentration dependent effect of streptonigrin on nuclear morphology in 3T3 cells (Fig. 2E). Higher concentrations of streptonigrin (10 uM, 1 uM) induced the formation of prominent heterochromatin foci at shorter incubation time while lower concentrations also caused change in nuclear morphology and brightness, albeit to lesser degrees.

Since streptonigrin is known to have strong cytotoxic effects at high concentration (>1 μM), we were interested in looking at the effects of low concentrations of streptonigrin. For this purpose, we treated NIH 3T3 cells with 1 nM of streptonigrin for different length of time up to 16 hours then and live stained with Hoechst 33342. We found that while 1 nM concentrations of streptonigrin caused an increase in fluorescence in 3T3 cells after four hours of treatment, with longer incubation times it caused more dramatic increases in fluorescence brightness and in the size of foci formed (Fig. 2F). Thus, streptonigrin promotes heterochromatin formation at a concentration as low as 1 nM and has greater effects with higher concentration and/or longer time of treatment.

### Streptonigrin promotes heterochromatin prior to affecting cell proliferation and viability

One goal of our screening effort is to identify drugs that can promote heterochromatin formation and suppress cancer growth with low cytotoxicity. We have previously shown that increasing heterochromatin formation can inhibit tumor growth without significantly impacting cell division and viability^16^, thus may represent a novel strategy of cancer treatment. Upon verifying the ability of streptonigrin to promote heterochromatin formation at a concentration as low as 1 nM, we investigated the cytotoxic effects of streptonigrin at different concentrations.

**Figure 3:**
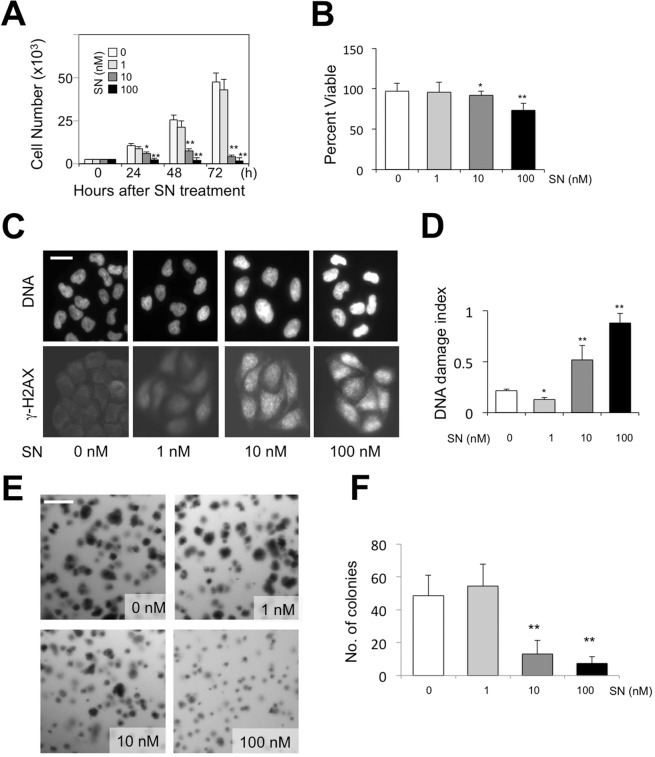
Effects of streptonigrin on proliferation, viability, growth of HeLa cells. (**A**) Cell proliferation assay of DMSO and streptonigrin treated HeLa cells. Cells were seeded at a density of 20,000 cells/well in 24 well plates and allowed to adhere for 12 hours. Cells were then treated with solvent (DMSO) only, 1 nM, 10 nM or 100 nM streptonigrin in DMSO for 24 hours. Cells were counted at 24, 48 and 72 hrs after initial exposure. Triplicate samples of each time point were counted by hemocytometer, averaged and plotted. Error bars represent s.e.m. * donates p < 0.05, **p < 0.01 by Student’s t-test. (**B**) Cell viability assay of DMSO and streptonigrin treated HeLa cells. Cells were seeded at a density of 25,000 cells/well in 24 well plates and allowed to adhere and grow for 36 hours. Cells were then treated with DMSO, 1 nM, 10 nM, or 100 nM streptonigrin for 48 hours. After treatment, media with dead, unadhered cells was collected and remaining cells were trypisinized. Viable cells were counted with trypan blue. Triplicates of each sample were counted and averaged. Error bars represent s.e.m. Scale bar = 5 μm. (**C**) DNA damage was assessed with antibodies specific for γ-H2AX. Cells were seeded at a density of 25,000 cells/well in 24 well plates and allowed to adhere and grow for 24 hours. Cells were then treated with solvent (DMSO), 1 nM, 10 nM, or 100 nM streptonigrin for 4 hours, and were then fixed and immuno-stained with anti-γ-H2AX and DAPI. (**D**) Immuno-stained cells described above (**C**) were photographed on a fluorescence microscope and the ratios of fluorescence intensity of treated cells vs DAPI were calculated at DNA damage index for different concentrations of streptonigrin treatment. (**E**) HeLa cells were seeded in soft agar/DMEM with the indicated concentrations of streptonigrin and were grown for three weeks. The culture was photographed and the number of colonies with >0.5 mm diameter were counted in in 10×10 mm fields. Representative images are shown. Scale bar = 100 μm. (**F**) Histograms of colonies with >0.5 mm diameter from three independent experiments are shown. Error bars represent s.e.m. ** indicates p < 0.01 by Student t-test.

To this end, we treated HeLa cells with 1 nM, 10 nM and 100 nM of streptonigrin, respectively, for 24 hours and evaluated cell proliferation and viability. We found that HeLa cells treated with streptonigrin at 1 nM showed no significant change, at 10 nM showed a dramatic decrease in cell counts by 72 hours, and that cells treated with streptonigrin at 100 nM showed a significant decrease in cell counts as early as 24 hours (Fig. 3A). These results suggest that streptonigrin at higher concentrations (>10 nM) are causing HeLa cells to enter cell death and detach from the culture plate. In contrast, cells treated with low concentrations of streptonigrin (1 nM) seem to proliferate normally.

To determine if the effects are reversible, we treated HeLa cells at different concentrations of streptonigrin for 4 hours and then washed (changing to drug-free media for three times) and observed cells 16 hours later. We found the phenotype (increased fluorescence) can be reversed after treating cells with 10 nM, but was not completely reversed with 100 nM or higher concentrations of streptonigrin.

We next assessed cell viability by seeding HeLa cells at a density of 25,000 cells/well into 24 well plates and allowed them to adhere and grow for 24 hours. They were then treated with streptonigrin at 1 nM, 10 nM, 100 nM, or solvent (DMSO) only for 48 hours. After treatment we counted and calculated the percentage of cells that were in late apoptosis with trypan blue, a negative stain that is positive for late apoptotic cells. Cell viability for DMSO, 1 nM, 10 nM, and 100 nM streptonigrin treated cells were 96.72%, 95.78% and 91.95%, 73.44%, respectively. We found that 10 nM and 100 nM streptonigrin treated cells showed a significant decrease in cell viability (Fig. 3B**)**. Using Student’s t-test, we found that p-values for 1 nM 10 nM, and 100 nM streptonigrin treated cells compared to DMSO control were 0.52, 0.023, 0.008, respectively. These experiments suggest that at concentrations >10 nM streptonigrin may primarily cause cell death, which consequently affects cell population.

To further understand how streptonigrin may cause cell death, we investigated whether the drug can cause DNA damage by examining the levels of phosphorylated H2AX (γ-H2AX), a commonly used DNA damage marker^30^. We found that treating HeLa cells for 4 hours with 10 nM or 100 nM streptonigrin caused significant increases in DNA damage, as evidenced by increased γ-H2AX levels (Fig. 3C). Interestingly, treating cells with 1 nM streptonigrin, which causes an increase in heterochromatin levels (see Fig. 2B–E), did not cause DNA damage, as judged by γ-H2AX levels, and there is even a decrease in γ-H2AX levels (Fig. 3C). Thus, while at higher concentrations (10 or 100 nM), streptonigrin appears to cause DNA damage, no obvious DNA damage was detectable when cells were exposed to low concentrations (1 nM) of streptonigrin, at which heterochromatin increase was detectable. In other words, streptonigrin appears to cause an increase in heterochromatin independent to DNA damage.

Finally, we investigated the effects of different concentrations of streptonigrin on the ability of cancer cells to grow and divide into groups using the colony formation assay^31^. Similar to its effects on cell proliferation, we found that while 10 or 100 nM streptonigrin significantly inhibited colony forming abilities of HeLa cells in soft agar, 1 nM of streptonigrin had no significant effects compared with control (Fig. 3E,F). Taken together, we have found that, while low concentrations (1 nM) of streptonigrin promotes heterochromatin formation (see Fig. 2), it has minimal effects on cell proliferation and viability, and these cellular properties are affected only at higher concentrations (>10 nM). These observations suggest that the primary effects of streptonigrin might be to induce heterochromatin formation.

### Stretptonigrin inhibits STAT3 phosphorylation at low concentrations

**Figure 4:**
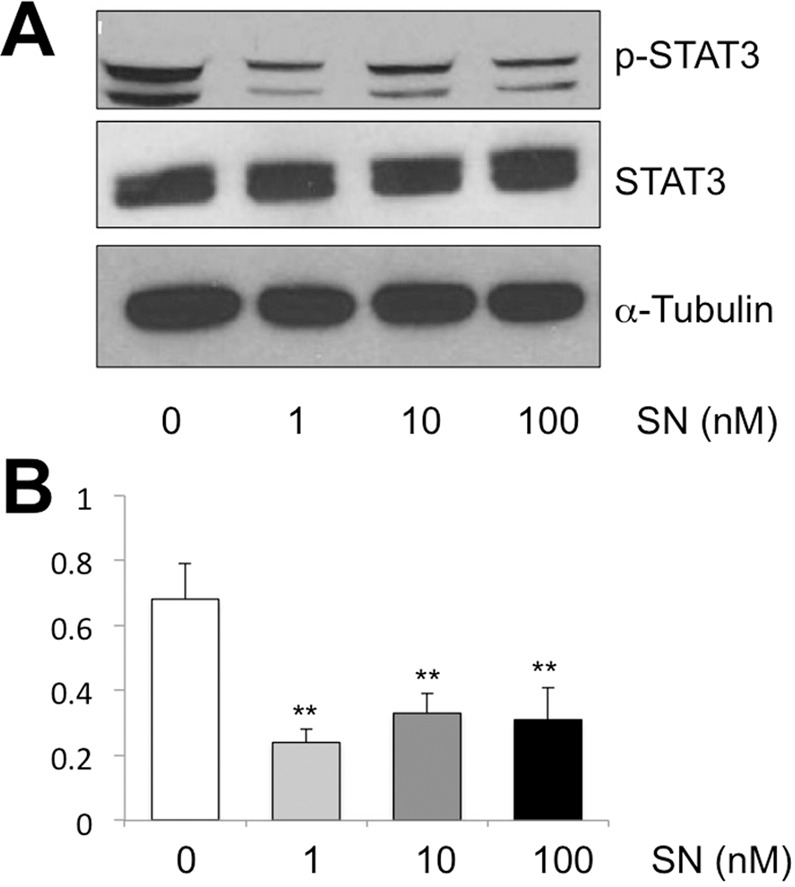
Streptonigrin inhibits STAT3 phosphorylation at 1 nM concentration. (**A**) HeLa cells were incubated in media with the indicated concentrations of streptonigrin or solvent (DMSO) only for 4 hours and were then homogenized and subjected to SDS-PAGE, and the protein was blotted with antibodies against pSTAT3, STAT3, and α-Tubulin, respectively. (**B**) Western blots were done in triplicates and the ratio of gel band intensities of pSTAT3 vs STAT3 were measured with Image-J and averaged. Error bars represent s.e.m. **indicates p < 0.01 by Student t-test.

We have previously shown a non-canonical STAT function, in which unphosphorylated (uSTAT) promotes heterochromatin formation and suppresses tumor growth^16,32,33^. Our recent drug screen for oncology drugs based on a *Drosophila* heterochromatin-based eye variegation phenotype has identified methotrexate as promoting heterochromatin formation^17^. It has been shown that methotrexate inhibits STAT phosphorylation^34^. Interestingly, streptonigrin has also been identified as an inhibitor of STAT phosphorylation in a drug screen^35^. We confirmed this finding and further found that streptonigrin can inhibit STAT3 phosphorylation in HeLa cells at concentrations as low as 1 nM (Fig. 4). Although the mechanism by which streptonigrin promotes heterochromatin formation is still under investigation and is beyond the scope of this paper, the results are consistent with, although do not prove, the idea that streptonigrin may act in conjunction with affecting STAT phosphorylation in promoting heterochromatin formation.

## Discussion

Heterochromatin formation is a cell-intrinsic tumor suppression mechanism^4,10,16^, and drugs that promoting heterochromatin formation with minimal cytotoxic effects are ideal as anticancer therapeutics. We have previously developed an *in vivo* screening method for identifying compounds that promote heterochromatin formation using *Drosophila* and have shown success in a small scale pilot screen, identifying methotrexate as a heterochromatin-promoting drug^17^. While *Drosophila* offers a convenient *in vivo* system, the screening procedure is relatively tedious and involves subjective judgment on the degree of red eye color as a measurement of heterochromatin levels. In order to develop a high throughput method for a streamlined process of screening for drugs that promote heterochromatin formation, we designed a simple screen, which is based on changes in fluorescence intensity of the cell nuclei stained with the DNA stain Hoechst.

Using HeLa cells, we screened two compound libraries provided by the NCI DTP with a total number of 231 drugs. Based on changes in fluorescent intensity and nuclear morphology in HeLa cell nuclei after drug treatment, we identified streptonigrin as a compound that induces heterochromatin formation. We further verified by using different means that streptonigrin is indeed able to promote heterochromatin formation. Finally, we established that streptonigrin at concentrations as low as 1 nM caused an increase in heterochromatin without significantly affecting cell proliferation and viability, raising the possibility that this drug might be useful as a cancer drug with low cytotoxic side effects. We are currently investigating this possibility.

Although we have shown that streptonigrin promtoes heterochromatin formation, the mechanism by which it does so, however, is not clear. We envision the following possibilities. First, we have previously identified a non-canonical STAT function, in which unphosphorylated STAT (uSTAT) promotes heterochromatin formation^16,32,33^. Interestingly, streptonigrin has been identified as an inhibitor of STAT phosphorylation in a phospho-flow single-cell drug screen using the NCI Natural Products drug library^35^. Coincidently, our recent *Drosophila*-based screen for heterochromatin-promoting drugs using the NCI Oncology library identified Methotrexate, which is also capable of inhibiting STAT phosphorylation^17^. Thus, streptonigrin could be inducing heterochromatin formation by preventing phosphorylation of STAT proteins. However, whether or to what extend inhibiting STAT phosphorylation accounts for the effects of streptonigrin and methotrexate on heterochromatin requires further investigation.

Second, streptonigrin, as well as methotrexate, has other cellular targets and the resulting cellular stress and/or DNA damage may induce heterochromatin formation as a protective mechanism. For instance, a group developed and performed a fluopol-ABPP HTS assay that identified streptonigrin as a potent, selective and irreversible PAD4 inhibitor^36^. PAD4, peptidyl arginine deiminase 4 is an enzyme that converts Arg or monomethyl-Arg to citrulline in histones and has been shown to be associated with chromatin decondensation in neutrophils^37^. The 7-amino-quinoline-5,8-dione core of streptonigrin has been identified to be a highly potent pharmacophore that acts as a pan-PAD inhibitor by examining a library of 32 analogues of streptonigrin^38^. While it is unclear whether streptonigrin’s ability to inhibit PAD4 is responsible for the increase in heterochromatin that we have observed, this library of analogues may prove useful into providing insights to the mechanism in which heterochromatin is being affected by streptonigrin. Further investigation is underway in order to understand the molecular mechanisms by which streptonigrin and methotrexate promote heterochromatin formation.

As we have previously demonstrated the ability of HP1 and unphosphorylated STAT5A to suppress cancer growth without significantly affecting the expression levels of genes that control cell cycle and apoptosis^16^, we also hope to verify that our drug hits have the ability to suppress cancer growth *in vivo* without severe cytotoxic side effects. Indeed, we have found that at low concentrations (e.g., 1 nM), streptonigrin is capable to increasing heterochromatin levels without affecting cell proliferation and viability. Establishing a low concentration that is capable of inhibiting cancer growth without cytotoxic effects could offer unique treatments that result in fewer side effects than those from current chemotherapy treatments.

Finally, we plan to use our cell-based screening method, which is inexpensive, easy to perform, and is high throughput to screen a larger number of compounds for heterochromatin-promoting drugs. We plan on using NIH 3T3 cells instead of HeLa cells for future screens, as 3T3 cells exhibit easily discernible foci, making detection of less drastic changes on nuclear morphology possible. Also, because of the discernible pericentric heterochromatin foci 3T3 cells possess, we may be able to quantitatively measure changes in heterochromatin organization using CellProfiler or other analysis software. We are currently in the process of screening the Mechanistic Diversity Set II, a set of 817 compounds provided by the DTP for more drugs that may promote heterochromatin formation.

## Methods

### Cell culture and screening method

HeLa human cervical cancer cell line and 3T3 immortalized mouse fibroblast cell line were cultured in Dulbecco’s Modified Eagle Medium (DMEM,) containing 100 μg/uL penicillin, 100 ug/μL streptomycin and supplemented with 10% (v/v) FBS.

To screen for heterochromatin-promoting drugs, HeLa cells were plated onto 96 well plates at a concentration of 7,000 cells per well in 180 μl media and allowed to adhere and grow overnight for 24 h. The next day, 20 ul of media with drugs were added to the wells to make a final concentration 10 μM in 200 μl media and allowed to incubate for 4 hours. The initial screening had to be done on fixed cells because imaging had to be done manually. After drug treatment, cells were washed with PBS then fixed in 100 μL 3.7% (v/v) formaldehyde for 15 minutes, permeabilized with 100 μL 0.3% PBT in PBS for 20 minutes and stained with 100 μL 1 μg/ml Hoechst 33342 in PBS +0.3% PBT for 15 minutes. 30 μL of 70% glycerol in ddH20 was then added to the wells for preservation.

For live staining and imaging, 3T3 cells were seeded at a density of 100,000 cells/well in 24 well plates and allowed to adhere overnight. Cells were then incubated with 1 nM, 5 nM, 10 nM, 100 nM, 1 μM, 10 μM streptonigrin or DMSO only for 4 hours. After drug treatment, Hoechst 33342 in DMEM was added to a final concentration of 10 μM. After 15 min of incubation, cells were photographed with a fluorescence microscope.

The cell images were analyzed using CellProfiler and its built-in modules to obtain mean fluorescence intensities for the drug treatment.

### Immunofluorescence and Western blotting

HeLa cells were plated onto 6 well plates at a concentration of 0.3×10^6^ cells/well and allowed to adhere overnight. The next day cells were treated with 10 uM streptonigrin for 6 hours. Afterwards cells were fixed with 500 ul/well 3.7% (v/v) formaldehyde for 15 minutes, permeabilized with 500 μl of 0.3% PBT and 1% BSA in PBS for 20 minutes. 500 ul of primary antibody against HP1α (or CBX5) (1:200; Life Sciences, 730019) was then spread and allowed to incubate overnight at 4 °C. The next day, cells were then incubated with secondary antibody at room temperature for 120 minutes, and mounted on coverslip with DAPI and observed under fluorescence microscope.

Primary antibodies used in immunostaining and Western blotting include mouse antibodies against HP1α (or CBX5) (1:200; Life Sciences, 730019), rabbit antibodies against H3K9me3 (1:500, MilliporeSigma (Burlington, MA), 07–442), goat anti-p-STAT3 (1:250, Santa Cruz, sc-7993), and rabbit anti-STAT3 (1:500; Santa Cruz, sc-482).

### Cell proliferation/viability assays

For cell proliferation assays, HeLa cells were plated onto 24 well plates at a density of 20,000 cells/well and allowed to adhere for 12 hours. Cells were then treated cell with 1 nM, 10 nM, 250 nM streptonigrin or DMSO for 24 hours. Cells were detached by trypsinization using 100 uL 0.05% trypsin-EDTA at 37 °C for 8 minutes and then trypsin was deactivated with 200 uL of DMEM. Cells were counted physically at 24, 48 and 72 hours after initial exposure using hemocytometer. Triplicates were done for each treatment and counts were averaged.

For cell viability assays, HeLa cells were seeded into 24 well plates at a density of 25,000 cells/well and allowed to adhere for 36 hours. Cells were then incubated with 1 nM, 5 nM, 10 nM streptonigrin or DMSO-only. Media pooled with detached cells was saved and remaining adhered cells were then detached by incubating cells with 100 uL of 0.05% trypsin-EDTA at 37 °C for 8 minutes, deactivated with 400 uL of DMEM and media with detached-cells was recollected. Viability was then assessed by diluting cells into 0.4% trypan blue in ddH20 and at least 300 cells in each sample were counted. Triplicates were done for each treatment and averaged.

### Fluorescence resonance energy transfer (FRET)

To detect changes in histone methylation (H3K9me3) levels in living cells, cells were transfected with a CFP/YFP histone methylation FRET reporter construct^26^, obtained from Addgene (Cambridge, MA; cat. 22866). Donor and acceptor bleed through was corrected using donor and acceptor only samples. FRET measurements were carried out using a Zeiss Axio Observer fluorescence microscope equipped with FRET setup and software.

### Anchorage-independent colony formation assay

HeLa cells were maintained in Dulbecco’s Modified Eagle’s Medium (DMEM) supplemented with 10% FBS. Cells grown to 60% confluency were suspended at a 1×10^5^ cells/mL in 0.4% Noble agar (Sigma) containing DMEM, and were placed on top of an underlayer of 0.8% agarose in DMEM in tissue culture plates. Cell media were refreshed twice weekly and the culture were maintained for 3 weeks in a tissue culture incubator. Colonies were stained with p-iodonitrotetrazolium (Sigma) and photographed on a microscope. The number of colonies >0.5 mm in diameter in a 10×10 mm field was counted and averaged for multiple plates.

## Supplementary information

Supplementary Information.

Supplementary Dataset 1.

## Data Availability

The authors will provide materials, data and associated protocols promptly to readers upon request.
