# Supplementary material for: Streptonigrin at low concentration promotes heterochromatin formation
Source: Sci Rep. 2020 Feb 26;10:3478. doi: 10.1038/s41598-020-60469-6 (PMC7044429; doi:10.1038/s41598-020-60469-6)
Supplement: Supplementary file 1 — Supplementary Information. [file 41598_2020_60469_MOESM1_ESM.docx]

**Supplementary Information**

**Streptonigrin at low concentration promotes heterochromatin formation**

Andre C. Loyola, Kevin Dao, Robin Shang, Lin Zhang, Pranabananda Dutta, Cody Fowler, Jinghong Li, & Willis X. Li*

Department of Medicine, University of California San Diego, La Jolla, CA 92093

*corresponding author (wxli@ucsd.edu)

Running title: Streptonigrin promotes heterochromatin formation

Keywords: Streptonigrin, heterochromatin, JAK/STAT, cancer, tumor suppression, drug screen

**Supplementary Figure Legends**

**
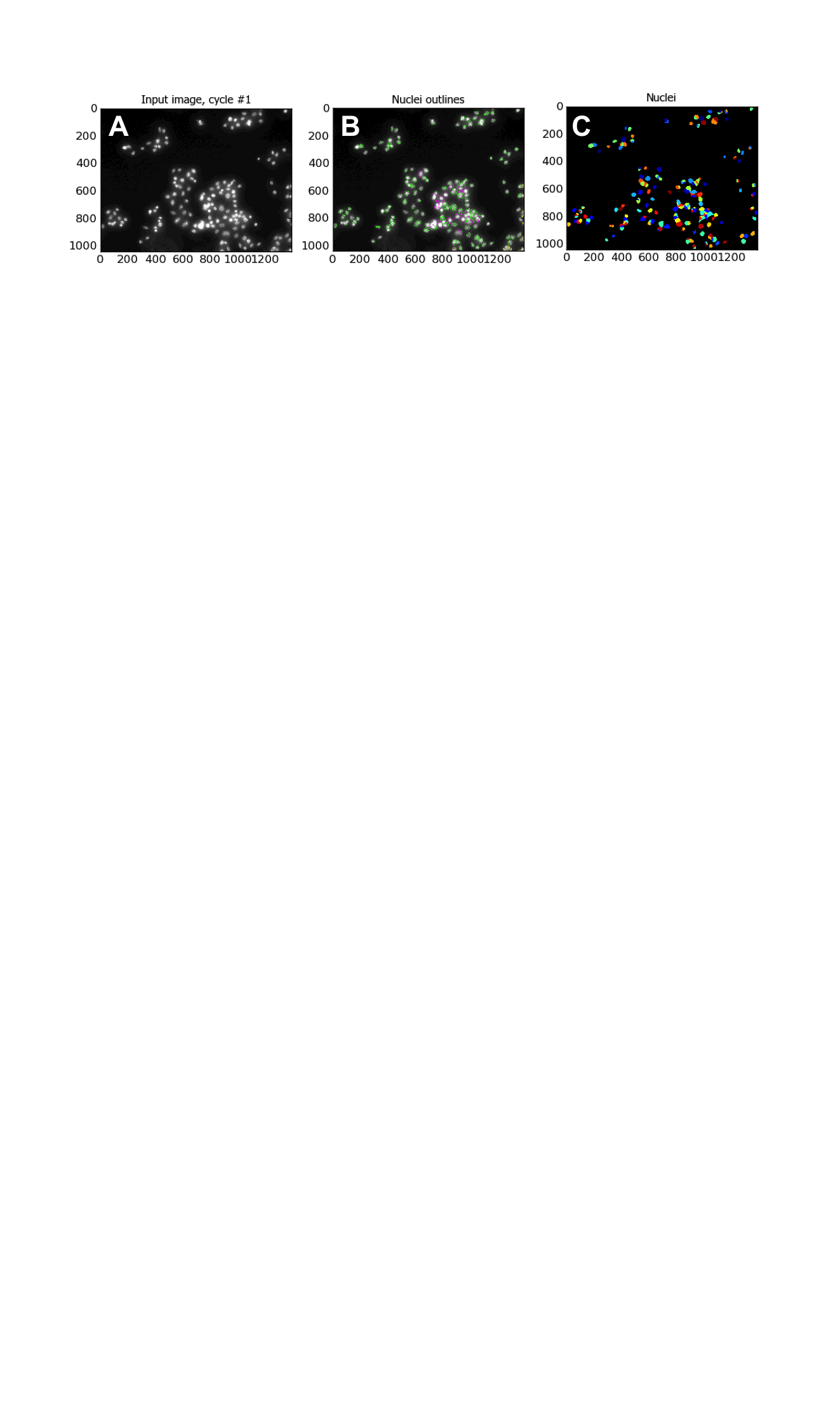
**

**Figure S1. Quantification of nuclear fluorescence intensity using CellProfiler**

**(A)** An image of HeLa cells treated with 1 nM streptonigrin and stained with Hoechst 33342 was used as an input image. **(B)** CellProfiler’s built-in object identification module was used to outline and identify the nuclei in each image. **(C)** A CellProfiler pile line was used to measure nuclear fluorescence intensity.


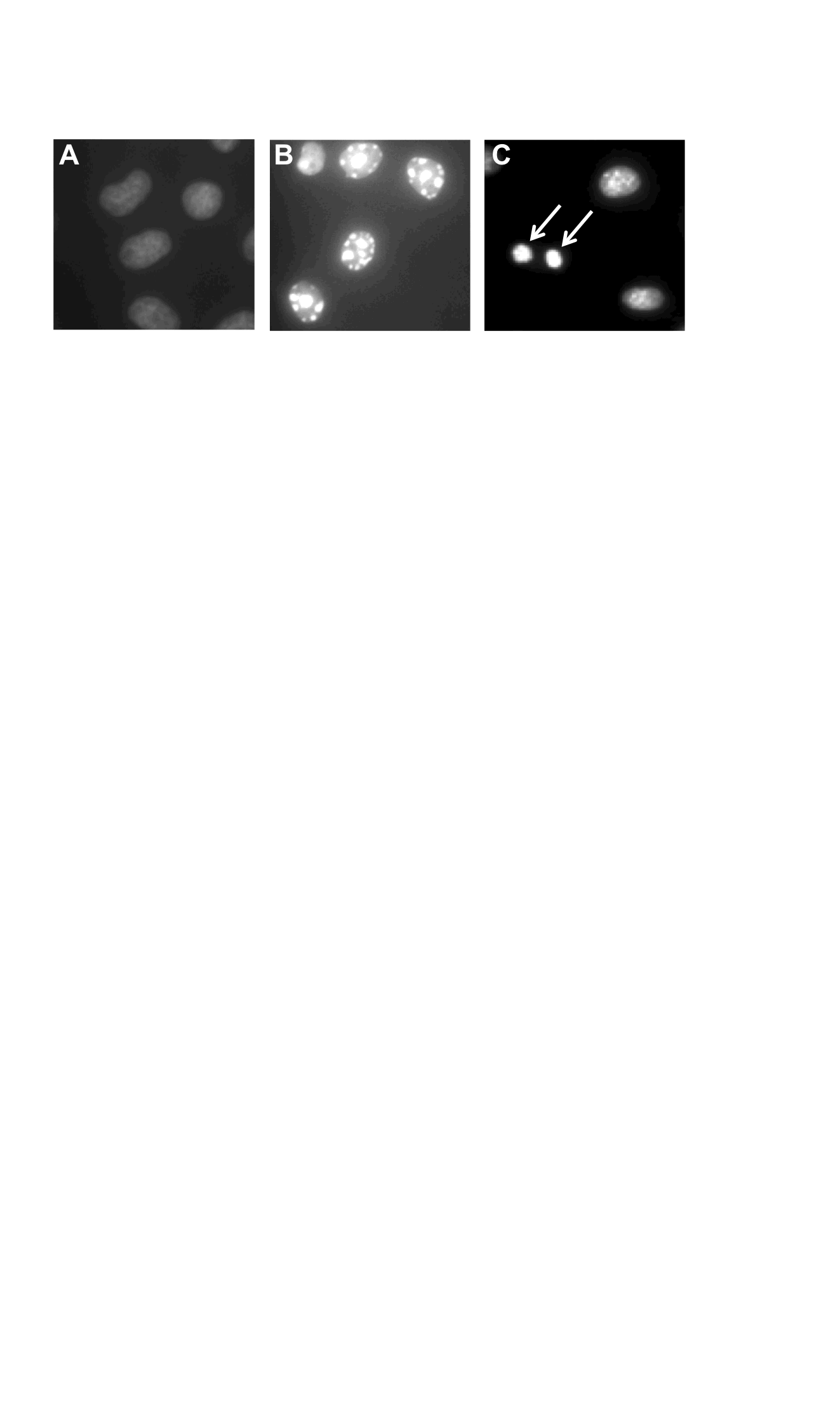


**Figure S2. Images of cell death**

HeLa cells cultured in DMEM and treated as the following and then stained with Hoechst 33342.

**(A)** HeLa cells were treated with solvent only (control).

**(B)** HeLe cells were treated with 10 µM streptonigrin for extended amount of time (overnight, >16 hours), resulting in cell death phenotypes including chromatin margination and nuclear fragmentation.

**(C)** HeLa cells treated as above showing nuclear condensation (arrow).
